# Comparison between stem cell therapy and stem cell derived exosomes on induced multiple sclerosis in dogs

**DOI:** 10.1186/s12917-024-03920-4

**Published:** 2024-03-08

**Authors:** Ahmed N. Abdallah, Ashraf A. Shamaa, Omar S. El-Tookhy, Mohamed M. Bahr

**Affiliations:** 1 Hormones Department, Medical Research and Clinical Studies Institute, National Research Center, Giza, Egypt; 2 Surgery, Anesthesiology and Radiology Department, Faculty of Veterinary Medicine, Cairo University, Giza, Egypt

**Keywords:** Spinal cord injury, Multiple sclerosis, Stem cell therapies, Bone marrow stromal cells, Exosomes, Platelet-rich plasma

## Abstract

**Background:**

Multiple sclerosis (MS) is a chronic condition that primarily manifests as demyelination of neuronal axons in the central nervous system, due to the loss or attack of oligodendroglia cells that form myelin. Stem cell therapy has shown promising results for the treatment of MS due to its capability to halt the immune attack, stop apoptosis and axonal degeneration, and differentiate into oligodendrocytes. Stem cell-derived Exosomes (Exosomes) have shown great capabilities for neuronal diseases as they have growth factors, complex sets of miRNA, enzymes, proteins, major peptides, lipids, and macromolecules with anti-inflammatory, angiogenesis, and neurogenesis activities.

**Methods:**

This study aimed to compare the healing properties of stem cells, against Exosomes for the treatment of an experimentally induced MS dog model. Dog models of MS received either a single treatment of stem cells or a single treatment of Exosomes intrathecally and the treatment process was evaluated clinically, radiologically, histopathologically, and electron microscopy and cerebrospinal fluid analysis.

**Results:**

showed marked amelioration of the clinical signs in both treated groups compared to the control one, magnetic resonance scans showed the resolution of the hyperintense lesions at the end of the study period, the histopathology and electron microscopy showed marked healing properties and remyelination in treated groups with superiority of the stem cells compared to Exosomes.

**Conclusions:**

Although stem cell results were superior to Exosomes therapy; Exosomes have proven to be effective and safe important actors in myelin regeneration, and their use in diseases like MS helps to stimulate remyelination.

## Highlights

Multiple sclerosis is a chronic condition that manifests as demyelination of neuronal axons.

Stem cell therapy has shown promising results for MS treatment by halting the immune attack.

Stem cell-derived Exosomes have shown great modalities for the treatment of neuronal diseases.

Superiority of the stem cells compared to Exosomes in means of differentiation into neuronal glial cells.

Exosomes have proven to be effective and safe important actors in myelin regeneration.

## Introduction

Multiple sclerosis, commonly known as (MS) is a life-threatening autoimmune disease where progressive demyelination of the central nervous system neurons occurs. Oligodendrocytes are the cells responsible for myelin production which cover and protect the axonal part of neurons that accelerate the conduction of nerve impulses. For anonymous reasons, the immune system raids the oligodendrocytes and the shielding myelin sheath. This disease starts with severe inflammation of the myelin sheath, and then remyelination occurs. Progressive breakdown of oligodendrocytes results in permanent cell loss, gliosis, and the remyelination process stops leading to irreversible loss to the axons [1].

The modern alternatives for MS treatment depend on regenerative therapy as a cell-based therapy. Numerous articles have confirmed that Mesenchymal Stem Cells (MSCs) were effective in the induction of regeneration, neuroprotection, and physiological recovery in MS mice models [2], and some human clinical studies [3].

MSCs unveil the power to trans-differentiate into neuronal cells which cause neurogenesis and neuroprotection. MSCs share with other types of Stem Cells (SCs) the property of promoting a neuroprotective and neurotrophic environment after spinal cord injuries [4]. The positive effect on neuronal repair and recovery was attributed to the trophic effects of the MSCs, rather than the cellular replacement at the site of injury [5].

MSCs have important immunomodulating properties that suppress in vitro B- and T-cell functions in addition to NK cells. MSCs decrease the propagation of both types of CD4 + and CD8 + T lymphocytes, and NK cells, but they did not have a similar impact on B lymphocyte proliferation [6].

The immunosuppressive properties of MSC are not explained but there are two main suggested mechanisms. The humoral mechanism, by the creation and production of soluble components and cell-to-cell contact-dependent mechanism [2].

The cutting-edge technique for MS treatment is cell-free therapy using extracellular vesicles EVs (Exosomes). Exosomes are tinny spherical vesicles (average diameter is 50 nm) [7] surrounded by a lipid bilayer membrane [8], which contain growth factors, complex set of miRNA, enzymes, proteins [9], major peptides, lipids, and macromolecules with anti-inflammatory, angiogenesis, neurogenesis activities [10, 11] [12]. stated that in culture, cortex neurons release exosomes [13]. stated that astrocytes also secret exosomes. Exosomes were detected in cerebrospinal fluid, (CSF) at the embryonic stage [14] and in adults [15], proposing that they could be crucial to a healthy CNS.

The exosome-mediated exchanges between neurons and glial cells lead to the outgrowth of neurons and cause neuron survival [16] through the transfer of micro-RNA-133b to neurons [17].

### Aim

This study aimed to compare the healing properties of Stem Cells against Exosomes generated from Mesenchymal Stem cells for the treatment of an experimentally induced MS dog model.

## Materials and methods

### Research plan

An approved protocol was received from the Faculty of Veterinary Medicine at Cairo University’s Institutional Animal Care and Use Committee (IACUC). Approval ID#: CU/II/S/23/16.

### Experimental animals

In this investigation, 36 Mongrel dogs of both sexes (2–5 years old) were employed. Dog Source is the official supplier of the Faculty of Veterinary Medicine, Cairo University. In order to rule out and exclude any canines with any nerve symptoms, including paralysis, paraplegia, tremors, paresis, lameness, head tilts, etc., all dogs had a pre-study assessment. All animals were singly housed in the department kennels and fed ad libitum a balanced dog ration. At the end of the experiment, the dogs are euthanized using high doses of the anesthetic sodium thiopental.

### Animal groups

According to the treatment material, animals were uniformly and randomly allocated into 3-main groups after induction of MS using Ethidium bromide as gliotoxin: Treatment of all groups started on day 14 post-induction making sure that all the clinical signs were seen on the animals.

#### Group 1

(Control group); treatment with saline 0.9%.

#### Group 2

(Stem cells Treated group); treatment using autologous adipose-derived SCs.

#### Group 3

(Exosomes treated group); treatment using Stem cell-derived Exosomes.

Each group was divided into 4 equal subgroups based on the observational window, which was 3, 7, 14, and 28 days after the start of the therapy.

Animals were euthanized humanely when testing was completed and sections of the spinal cord were harvested for histopathological and electron microscopy (EM). The entire spine (T11-L3) was fixed in 10% Neutral Buffered Formalin for 24 h, after which the spinal cord was extruded from the bone, divided into 2 samples, and coronal spinal sections of 1 mm were taken for histopathological analysis using customary H&E stain on 10% Neutral Buffered Formalin as a preservative and for EM on 5% glutaraldehyde.

#### Induction of demyelinating lesions

The dorsal lamina of the first lumbar vertebra was punctured bilaterally with a dental drill while the dogs were under general anesthesia. The lateral columns of the spinal cord were injected with a 20-µl dose of ethidium-bromide concentration 0.1% using a microneedle syringe connected to a capillary tube [18].

#### Stem cell preparation

Under general anaesthesia, an aseptically created (2–3 cm) abdominal skin incision. Each dog’s autologous S/C fat (15–20 g) was extracted in sterile vessels. Chopped and thoroughly cleaned with phosphate-buffered saline to eliminate any debris or free blood, the obtained fat was then added to an equivalent amount of 0.1% collagenase type 1 (Sigma, Aldrich). The tissue was placed in an incubator that was constantly shaking at 37 °C for one hour. After assimilation, lipoaspirates were separated from collagenase by centrifuging for 10 min at 1200 rpm, and any enzyme that remained was washed away three more times. A hemocytometer was used to count the cells in the aspirates after the final round of centrifugation, and the trypan-blue-dye exclusion test was used to determine the viability of the cells [19]. When cells reached 70–80% confluency, they were trypsinized and divided to increase their number and were regarded as the first passage. When cells reached the third passage, they were harvested after trypsinization and characterized using flow cytometry markers CD90, CD 73, CD105, and CD 34. Cells were seeded in tissue culture flasks in DMEM low glucose media (Gibco) with 10% Fetal Bovine Serum, and the media was changed every three days [20]. Then, each case received an injection of the stem cell preparation (10 × 10^6^ cells in normal saline).

#### Exosomes

After the third passage, MSCs were grown in DMEM low glucose, which was supplemented with 0.5% of bovine serum albumin and was devoid of foetal bovine serum (Sigma). After two days of incubation the media was collected and centrifuged at 10,000 rpm for 10 min to remove any cell debris then the supernatants were collected and filtered using a 0.22 μm syringe filter. After that, the medium underwent ultracentrifugation at 100,000 rpm for 1 h in order to collect the Exosomes pellet. By using the Bradford test to measure protein content, 200 g/ml of exosomes were introduced to the sterilized Carboxymethylcellulose (CMC) at a concentration of 22 mg/ml. The gel contains 200 µg of exosomes per 1 mL [21]. Exosomes produced from MSCs in CMC gel and maintained by lyophilization, a technique suitable for preserving materials that are thermo-liable. Exosomes are readily preserved in a form that is continually storable, and they are quickly reconstitutable by merely adding water [11]. 

#### Injection route

Normal saline, Stem cells, or Exosomes were injected directly into the CSF through the foramen magnum in the proximal spinal cistern on the day-14 from the induction of the MS under digital X-ray guidance with a total volume of 2mls after aspirating an equal volume of CSF [18].

### Evaluation

#### Clinical evaluation

According to the Olby scale score chart, clinical alterations in gait, movement, sensation, reflexes, and proprioception were noted and assessed [22] in each of the evaluation periods.

#### Magnetic resonance

A 1.5 Tesla closed MR device was used for the MRI. MR Sagittal, dorsal, and transverse T2-weighted, T1-weighted, and sagittal STIR (TR/TE/TI 3310/61/140 ms) sequences were used for the imaging of the spine. From T11 to L3, the sagittal, dorsal, and transverse spinal sequences were carried out (vertebral body).

#### CSF analysis

CSF samples collected before treatment and at the end of the experimental study were evaluated chemically for the color, turbidity, glucose, chloride, proteins and bacteriologically measuring total leukocytic count, and differential leukocytic count, gram staining and Ziehl Nielsen Stain [23].

#### Histopathological evaluation

For light microscopy, tissue samples were fixed in paraffin blocks, cut into 10-m-thick slices, and then stained with H&E [24]. White matter and MS lesions were scored according to the following arbitrary scales: (0) no remyelination, (1) little remyelination at the lesional border, (2) significant remyelination, either confluent or patches, and (3) practically full or complete remyelination (shadow plaque).

#### Electron Microscopy

Semi-thin (0.5µ to 1µ) slices were prepared to determine the degree of demyelination and axonal loss, sections were prepared using an LKB® ultra-microtome, stained with toluidine-blue, and photographed using an SC30 Olympus® camera. Next, ultrathin Sects. (500–700 A) were created using a Leica AG ultra-microtome, contrasted with uranyl acetate and lead citrate, and scanned using a JEM-100-CXII.

### Statistical analysis

Data were summarized as means ± standard errors (SE). The effect of intervention was analyzed within each group using the Wilcoxon Signed Ranks Test. Differences between the treatment groups were tested using the One-way analysis of variance (ANOVA) test and Tukey post hoc test. Results were statistically analyzed using PASW Statistics Software, Version 18.0, from SPSS Inc. (Chicago, IL, USA). Significance was considered at *P* < 0.05.

## Results

On day 13 after induction, animals were examined (i.e., 1 day before commencing the treatment). All of the animals displayed clear signs of ataxia, including faulty steps, pelvic limb crossing, standing and walking on the dorsum of the foot, reduced tail movement, and extremely sluggish proprioception reflexes. Animals were given predetermined dosages of normal saline, stem cells, or exosome preparation on day 14.

### Clinical evaluation post-treatment

**Fig. 1:**
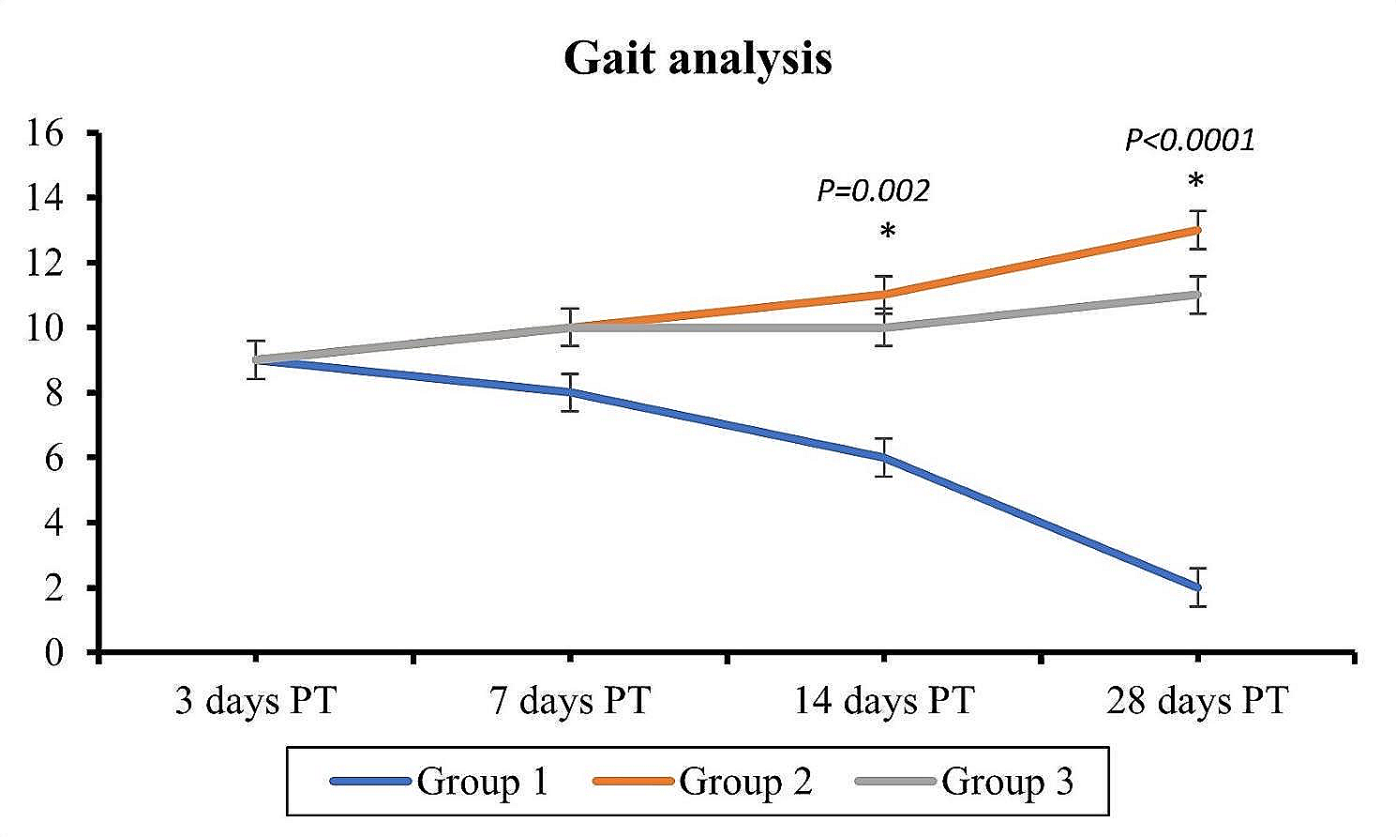
Showing the clinical gait evaluation scores over time. Data expressed as mean values. PT: post-treatment

After 3 days following treatment, the animals in all groups continued to exhibit ataxic gait with full weight bearing, an increase in the frequency of incorrect steps, and slow proprioceptive responses. The control animals required assistance to stand after 7 days because they had a noticeable rise in ataxic gait and a loss in pelvic limb strength. The ataxic gait of the animals treated with stem cells and exosomes was slightly improved, and they were able to walk alone with good pelvic limb strength but with still weak proprioception reflexes and tail motions. After 14 days, control dogs were not able to stand on their pelvic limbs due to decreased pelvic strength and muscle mass, as well as very slow reflexes. In contrast, the groups that had received treatment with stem cells and exosomes displayed significantly improved gait, flawless proprioception reflexes, and only weak tail movements. The control animals were essentially paralyzed after 28 days, unable to control their bowel and bladder movements and without any feeling or reflexes in either of their rear limbs. The Exosomes treated group showed a noticeable improvement in gait with full weight-bearing but with many faulty steps and limb crossing. The Stem cells treated group showed normal gait in all animals with almost no faulty steps and good tail movements, and the overall condition of the animals improved with a playful and jumping demeanor. Figure 1 depicts the variations in clinical gait across the groups using the Olby scale grading method.

### Magnetic resonance

**Fig. 2:**
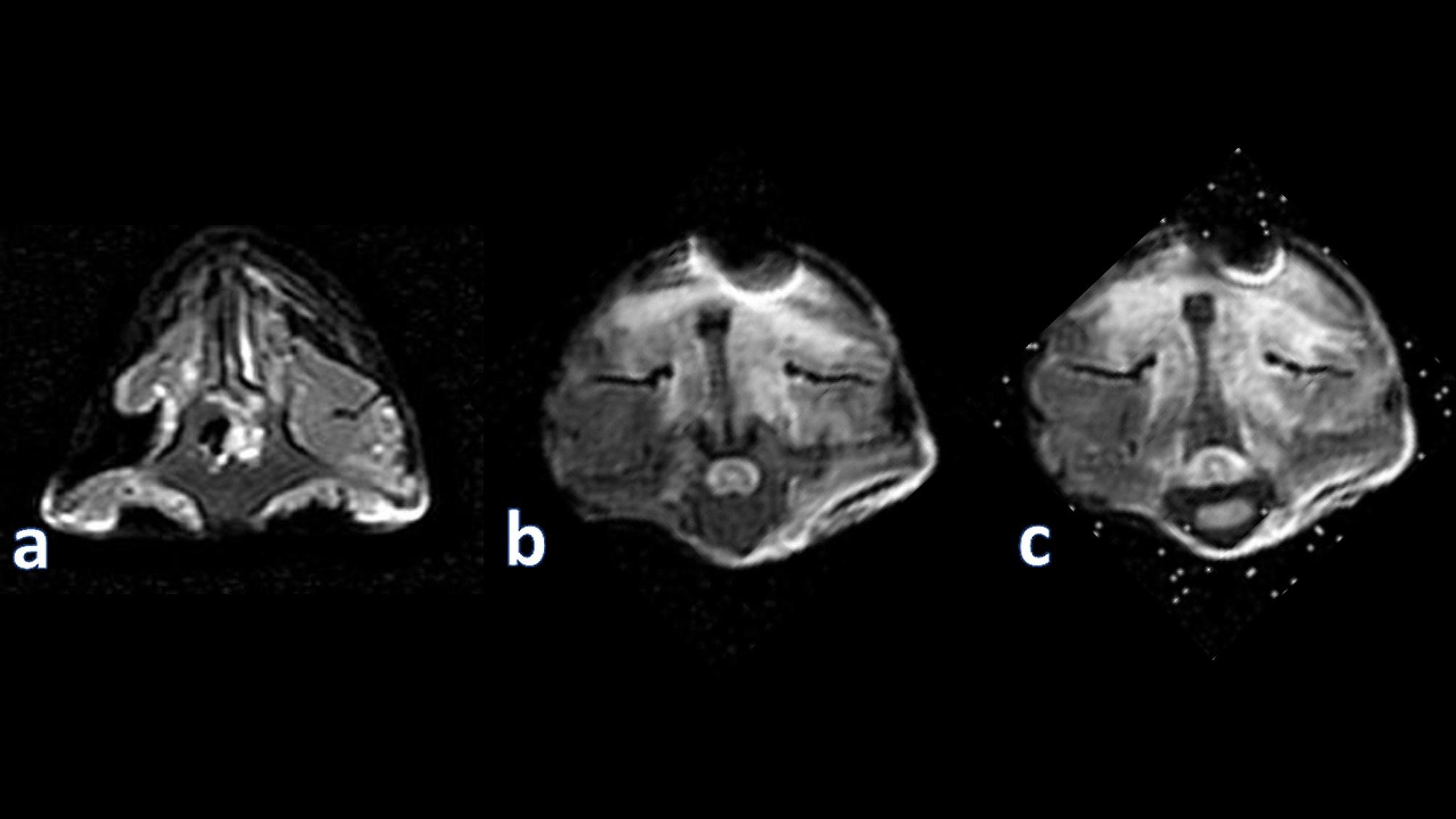
Showing MRI images of the spinal cord 28 days post treatment where **(a)** represents the control group; **(b)** represents the stem cell group showing normal scan of the spinal cord; **(c)** represents the Microvesicles group showing normal scan of the spinal cord

MRI scans of the treated groups’ spinal cords revealed faster recovery than in the control group. On T2 weighted images from all scans, there were bilateral hyperintense lesions, and on sagittal sections, there was a diffuse hyperintense lesion that was identified as syringomyelia. 3 days after the induction, whereas 7 days later, the treated groups’ MRI scans of the spinal cord exhibited less regions of hyperintensity on axial scans and sagittal sections. Large regions of hypointensity were visible on T1 weighted images on axial scans after 14 and 28 days, although treated subgroups of the same periods had normal intensity on both sagittal and axial scans with no aberrant findings (Fig. 2). Figure 2: showing MRI images of the spinal cord 28 days post-treatment.

#### CSF analysis

CSF analysis showed values within the normal range in all groups before and after treatment and no bacterial contaminants were isolated (Table 1).

**Table 1:** Showing the mean ± SE values of the CSF analysis of dogs before and after the end of treatment

	Control Group		Stem cell group		Microvesicles group	
	Before	After	Before	After	Before	After
Aspect	Clear	Clear	Clear	Clear	Clear	Clear
Color	Transparent	Transparent	Transparent	Transparent	Transparent	Transparent
Total Leukocytic count (cells/cmm)	10.0 ± 0.6	12 ± 0.6	9.0 ± 0.6	13.0 ± 0.6	10.0 ± 0.6	14.0 ± 0.6
Glucose (mg/dl)	70.5 ± 1.2	62.2 ± 1.2 ^B^	67.7 ± 1.2	80.1 ± 1.2 ^A^	67.8 ± 1.2	78.5 ± 0.7 ^A^
Chloride (mmol/l)	133.1 ± 0.5 ^a^	122.7 ± 0.5 ^C^	131.4 ± 0.5 ^a^	135.1 ± 0.5 ^A^	125.7 ± 0.5 ^b^	127.6 ± 0.5 ^B^
Proteins	30.0 ± 0.6 ^a^	25.0 ± 0.6 ^C^	23 ± 0.6 ^b^	27.0 ± 0.3 ^B^	25 ± 0.6 ^b^	30.0 ± 0.6 ^A^
Gram Stain	Negative	Negative	Negative	Negative	Negative	Negative
Ziehl Nielsen Stain	Negative	Negative	Negative	Negative	Negative	Negative

^a,b^ Different small letters superscripts indicate significance between different groups before treatment (Tukey test; *P* < 0.05). ^A,B,C^ Different capital letters superscripts indicate significance between different groups after treatment (Tukey test; *P* < 0.05)

### Histopathological evaluation

**Fig. 3:**
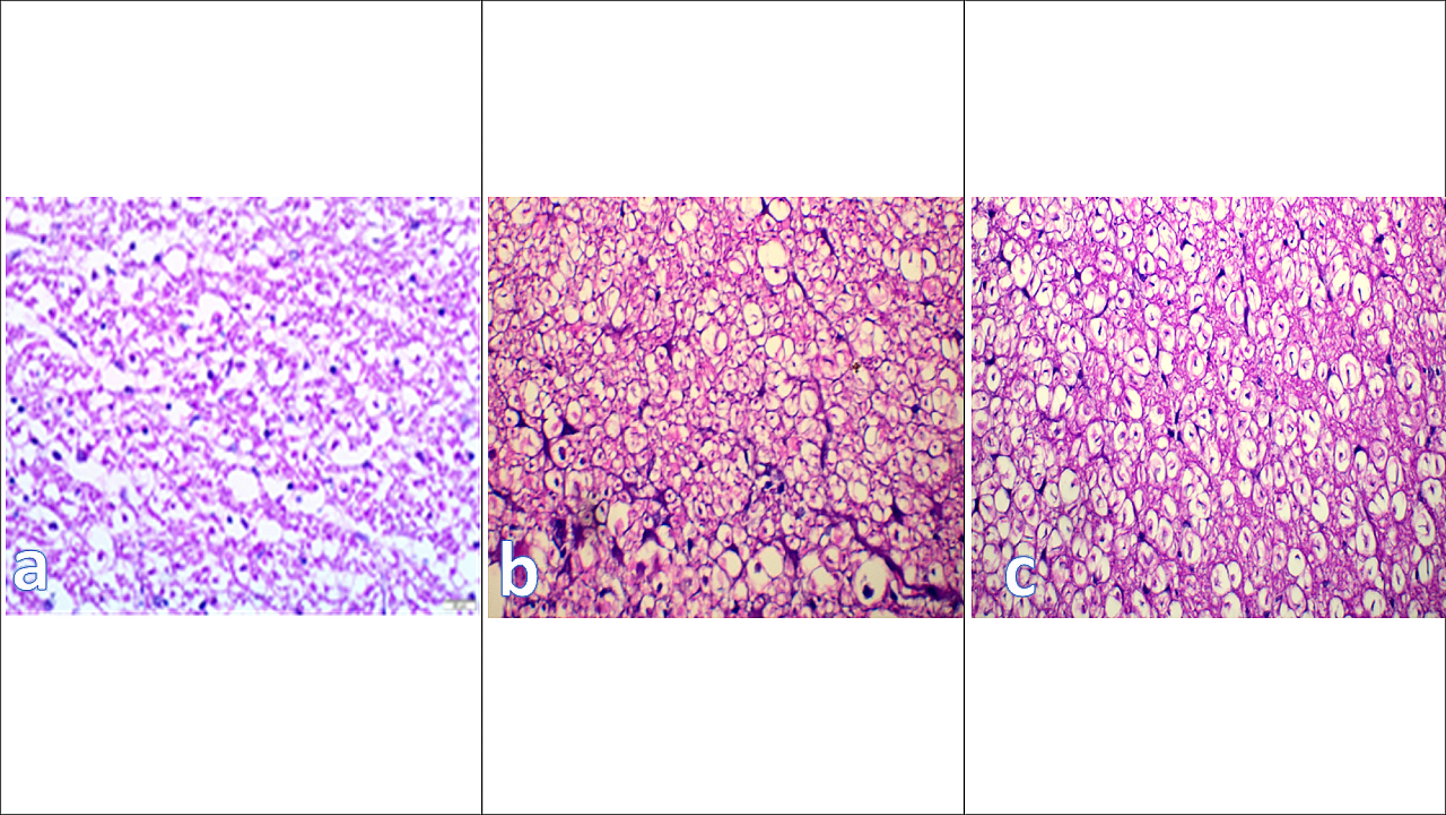
Showing histopathological evaluation of the spinal cord at 28 days post treatment where **(a)** the control group showing mass demyelination and vacuolation and axonal shrinkage; **(b)** stem cell group showing extensive areas of demyelination around large deeply stained cells; **(c)** microvesicles group showing preserved axonal shape and large areas of remyelination

All animals’ histopathology samples taken 3 days after treatment showed well-defined areas of vacuolations, and the white matter displayed a higher degree of Wallerian degeneration, which was manifested as complete demyelination around axons, axonal degeneration, and increased glial cell proliferation with distinct astrocytic swelling. A few macrophages (Gitter cells) with foamy cytoplasm engulfing the degraded myelin and axons and leaving a distinct vacuole were visible in the control group after 7 days. In the grey matter, the neuronal injury was shown as nuclear damage accompanied by chromatorhexis, chromatolysis, and neuronophagia. The stem group demonstrated the presence of large cells with deeply stained nuclei on the white matter’s periphery, the restoration of axons to their normal architecture, and the emergence of patches of remyelination; a similar picture was seen in the Exosomes group but with fewer cells present on the periphery. The control samples demonstrated an increase in vacuolated regions with sizable areas of Wallerian degeneration, axonal swelling, and axonal degeneration after 14 days. In contrast to the stem cell treated group and the Exosomes group, where remyelination activity developed as patches around the aforementioned big cells, the limited proliferation of Gitter cells and low phagocytotic activity were found with a significant degree of loss of architecture. Additionally, the control group had extensive regions of mass demyelination as well as additional areas of loss of architecture, vacuolation, and axonal degeneration 28 days after treatment. In contrast, more remyelination occurred in the stem cell-treated group than in the Exosomes group, and the majority of the axons recovered their myelin coating and axon form in the majority of the white matter (Fig. 3). Figure 3: showing histopathological evaluation of the spinal cord at 28 days post treatment.

**Fig. 4:**
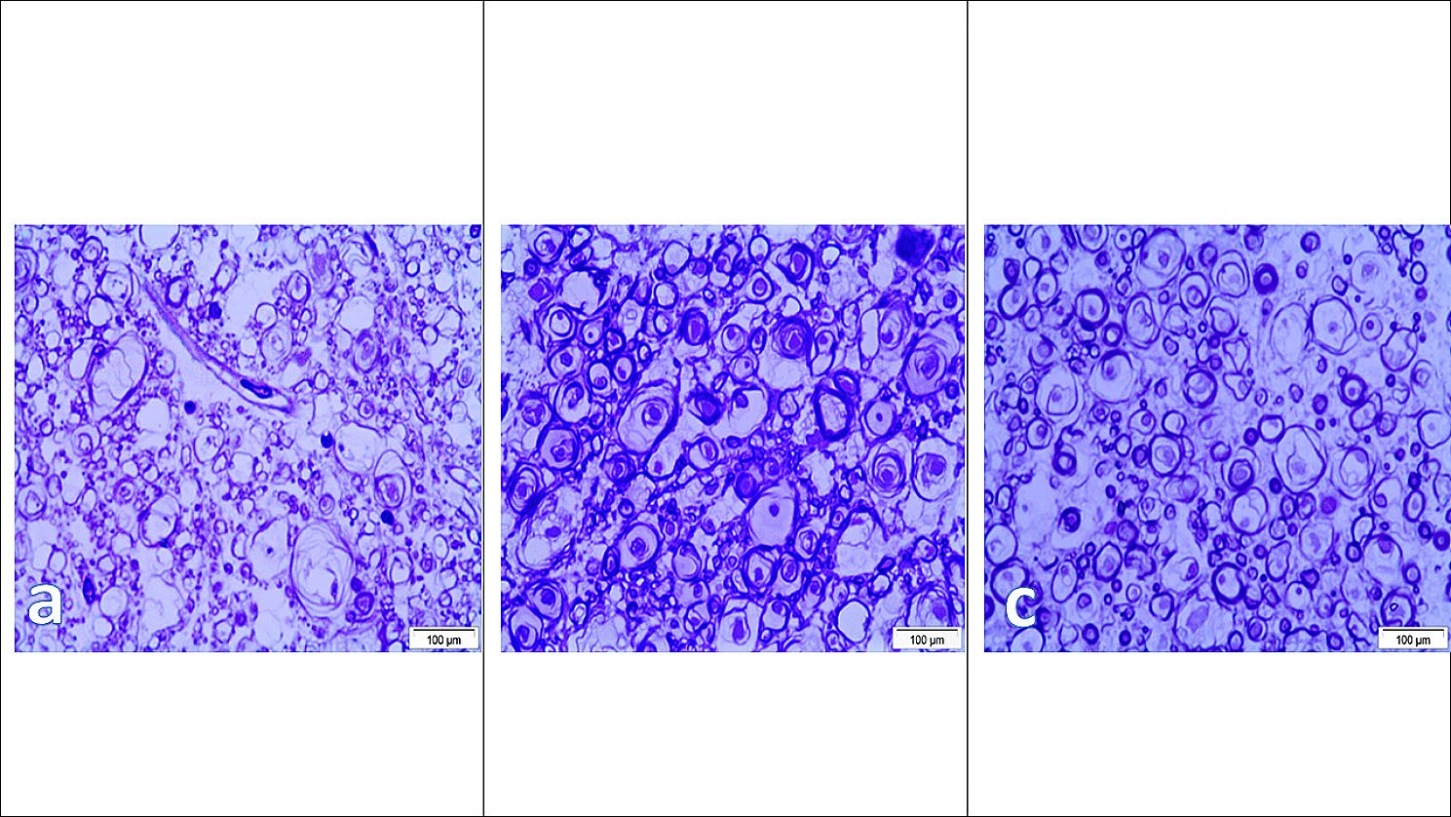
Showing toluidine blue stained spinal cord sections where **(a)** control group showing extensive signs of demyelination, axonal degeneration and vacuolation; **(b)** stem cell group showing marked signs of newly formed myelin sheaths around axons; **(c)** Microvesicles group showing the newly formed myelin around axons

Sections stained with toluidine blue were used to measure the degree of demyelination and axonal loss. After three days, the control group’s nerve fibers exhibited vacuolation or splitting of the myelin lamella along with axon shrinkage. Gliosis or a mild inflammatory cell response was seen. Semi-thin slices of the white matter from 7 to 14 days later showed vacuolation or splitting of the myelin lamella as well as shrinking of the nerve axons, as well as diffusely demyelinated nerve fibers. This became more pronounced after 28 days, at which time the white matter displayed pronounced deformity of the nerve fibers along with severe demyelination and axonal degeneration, shrinkage, folding, splitting, and/or vacuolation. A few nerve fibers underwent full destruction. As opposed to that, after 7 days post-treatment, both the stem cell group and the Exosomes group noticed a few cells with large vesicular nuclei containing one or two nucleoli and faintly stained cytoplasm in the interstitial tissue. These cells were associated with patchy remyelinating activity with varying degrees of staining affinity in the stem cell group but were not seen in the Exosomes group, which increased 14 days post-treatment as large patches of myelinated healthy Most of the axons had remyelinated after 28 days, and both groups’ big axons were covered in a heavily stained myelin sheath (Fig. 4). Figure 4: showing toluidine blue stained spinal cord sections.

### Electron microscopy

**Fig. 5:**
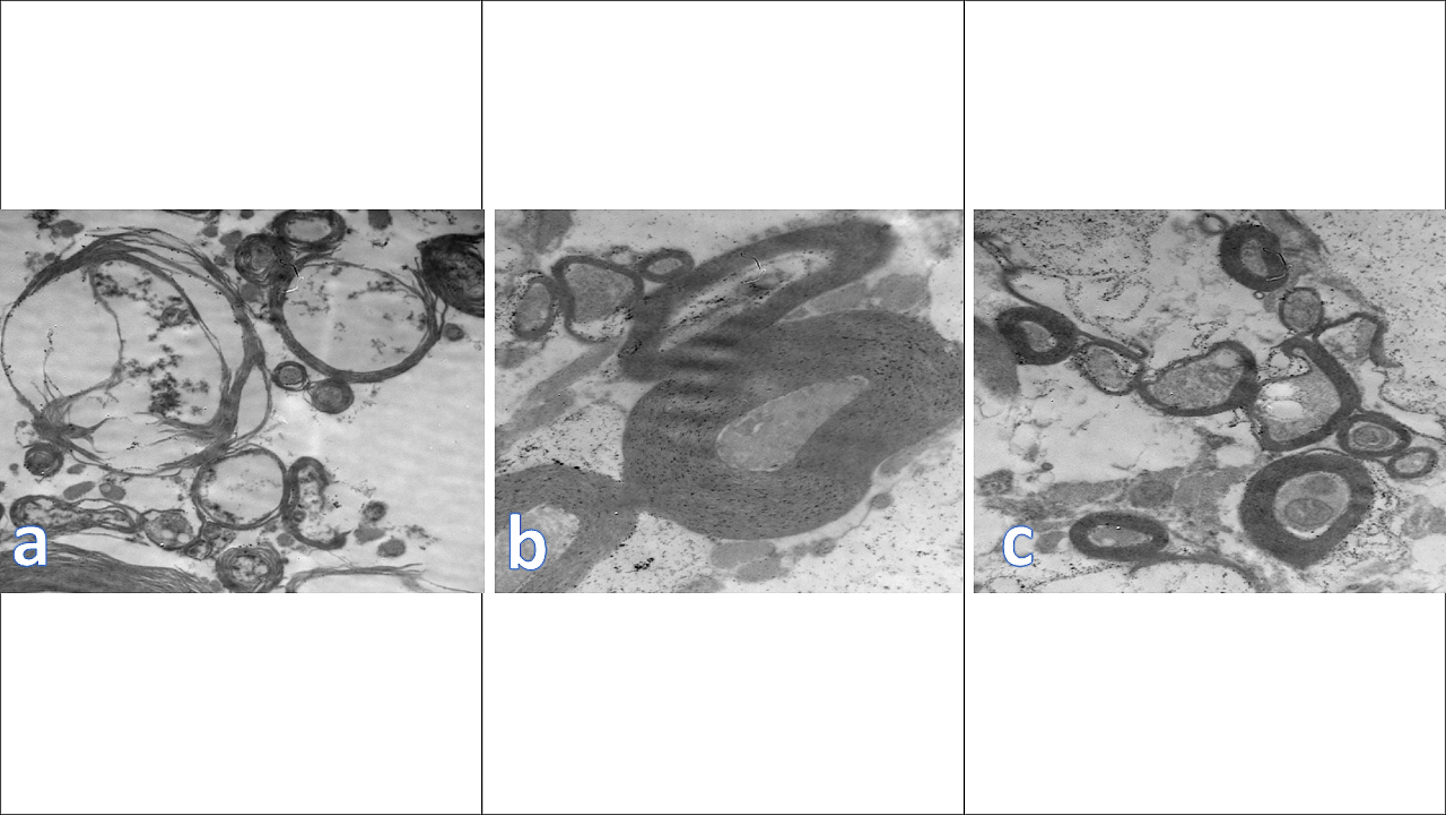
Showing electron microscopic analysis of the spinal cord sections where **(a)** represents the control group showing vacuolation, disintegration and axonal degeneration; **(b)** represents the stem cell group showing newly formed condensed myelin sheath of 1.50-micron thickness around normal axon **(c)** represents the microvesicles group showing normal myelin sheath and normal axons

The treated group’s spinal cord underwent electron microscopy analysis, and it was evident that three days after treatment, the axons were exhibiting a variety of deformities, including shrinkage, destruction, vacuolation, and degeneration. The myelin sheaths were also separated and destroyed, and phagocytic cells were engulfing the destroyed nerve fibers and sheaths. The astroglia and oligodendroglia also were phagocytosed; The myelin sheath was still immature, with the outer lamellar layer condensed and electron dens while the inner layer appeared light electron dens, folded and dispersed, leaving variable irregular size spaces between, in contrast to the other groups, which demonstrated large areas of active remyelination 7 days after treatment adjacent to large cells with a vesicular nucleus (N) and abundant cell organelles like free ribosomes, endoplasmic reticulum Nearly all of the axons had an electron-dense myelin sheath around them at 28 days after treatment, with high consistency. At 14 days after treatment, the majority of the axons had reverted to their natural architecture (Fig. 5). Figure 5: showing electron microscopic analysis of the spinal cord sections.

### Statistical analysis

The Wilcoxon signed-rank test results showed that each of the treatment courses did not elicit a statistically significant change in dogs before and after treatments (*P* > 0.05).

The one-way ANOVA revealed that there were statistically significant differences in glucose (*P* < 0.0001), chloride (*P* < 0.0001), and protein (*P* = 0.001) levels between the groups after treatments. However, total leukocytic count showed no significant differences between the groups (*P* = 0.125) (Table 1).

The one-way ANOVA revealed that there were statistically significant differences in gait between the groups at days 14 and 28 post-treatment (*P* = 0.002 and < 0.0001, respectively), where groups 2 and 3 were significantly higher than group 1 **(**Fig. 1**)**.

## Discussion

Stem cell therapeutics presents hope for the healing of neurological disorders like MS, due to its radical role in supporting neurons and axons; and also, differentiation capabilities into various neural lineages. While the main cause of MS is the loss of Myelin and disintegration of oligodendrocytic cells that are responsible for the formation and maintenance of Myelin sheath in the CNS, MSCs were proven to differentiate into oligodendrocytes, stopping apoptosis, and secreting trophic factors that stimulate the endogenous regeneration and halt the immune attack [18]. The biggest drawback of stem cells is the immunological danger, and since MSC implantation and differentiation into tissue cells cannot explain the functional repair of injured tissues that has been observed, there is growing evidence of another mechanism for tissue regeneration. It is well acknowledged that MSCs release a wide range of physiologically active cytokines/growth factors, extracellular matrix proteins, and tissue remodeling enzymes that are crucial for several tissue functions, repairs, and homeostasis processes. This secretome is known as exosomes and is currently used and evaluated for the treatment of various diseases including neurological disorders [25].

This study aimed to compare the effects of lyophilized ready-to-use canine exosomes derived from stem cells for the treatment of MS with actual stem cell therapy. Results showed that the injection of stem cells or Exosomes intrathecally is safe and did not induce any biochemical changes in the CSF composition which agrees with previous studies. The clinical evaluation of the treatment progress showed amelioration in gait and movements of both treated groups compared to the control with a better score in the stem cell treated group. Magnetic resonance of the animals showed a greater amelioration in treated groups compared to control one regaining the normal intensity of the spinal cord and resolution of the previous lesions which proves that stem cell therapy and/or Exosomes as cell-free therapy can ameliorate the signs of MS which can be noticed clinically and proved radiologically [26, 27]. The histopathological analysis and electron microscopy came explanatory to the previous results where the treated groups showed patches of active remyelination that increased over time accompanied by the appearance of large deeply nucleated cells near these areas of remyelination which were greater in number in the stem cell group which might be attributed to the direct differentiation of the injected cells into oligodendrocytes progenitors compared to the Exosomes group which only stimulated the proliferation of endogenous oligodendrocyte progenitors present in the spinal cord as stated by [28] who demonstrated that Exosomes from stem cells were picked up by oligodendrocyte progenitor cells, preventing them from going into apoptosis and encouraging cell survival, growth, and motility. These exosomes carried microRNAs and adhesion molecules that were the cause of the effects that were seen [29, 30].

Although stem cell results were superior to Exosomes therapy; Exosomes have shown that they are essential for myelin regeneration, and they may be used in conjunction with EVs to stimulate remyelination in pathological conditions [31].

## Conclusion and recommendation

In contrast to stem cell therapy, the use of exosomes is emerging as a more promising, safe, and practical strategy to immunomodulate, promote myelin regeneration, and conserve minimum immunological danger, therefore reducing neurodegeneration and ultimately improving patients’ outcomes. These results could pave the way for brand-new cell-free therapies to stop CNS chronic diseases.

## Data Availability

All data are available (data transparency) upon request.

## Abbreviations

MS: Multiple sclerosis
MSCs: Mesenchymal stem cells
SCs: stem cells
NK: Natural killer cells
EVs: Extracellular vesicles
CSF: Cerebrospinal fluid
CNS: central nervous system
EM: electron microscope
DMEM: Dulbecco’s Modified Eagle Medium
CMC: carboxymethyl cellulose
MRI: Magnetic resonance imaging
